# Chaihu Longgu Muli Decoction inhibits chronic stress-induced lung cancer epithelial-mesenchymal transition process by suppressing Rap1/ERK signal pathway

**DOI:** 10.3389/fphar.2025.1644315

**Published:** 2025-07-25

**Authors:** Zibo Li, Ziyang Huang, Zhiyi Wang, Zhenzhen Guo, Baoying Wang, Yucheng Li, Erping Xu

**Affiliations:** ^1^ Collaborative Innovation Center of Research and Development on the Whole Industry Chain of Yu-Yao, Henan Province, Henan University of Chinese Medicine, Zhengzhou, China; ^2^ Academy of Chinese Medical Sciences, Henan University of Chinese Medicine, Zhengzhou, China; ^3^ Henan Province Engineering Research Center of Zhang Zhongjing Classic Prescriptions Analysis and Development, Nanyang Normal University, Nanyang, China

**Keywords:** Chaihu Longgu Muli Decoction, chronic stress, lung cancer, Rap1, epithelial-mesenchymal transition

## Abstract

**Background:**

Chaihu Longgu Muli Decoction (CLM) is a classical herbal formula originally documented in *Shang Han Lun*. With an 1800-year clinical history, CLM remains widely prescribed for depression (“Yu Zheng” in Traditional Chinese Medicine theory). Emerging evidence suggests that chronic stress-induced depression is closely linked to lung cancer progression and metastasis. However, the therapeutic potential of CLM in this context remains unexplored.

**Methods:**

A lung cancer cell xenograft model combined with chronic unpredictable mild stress (CUMS) was used to evaluate the effect of CLM on lung cancer growth. Proteomic analysis was performed to explore the underlying mechanisms by which CLM alleviates CUMS-induced lung cancer progression. Western blot and qPCR were conducted to detect changes in Rap1/ERK-mediated epithelial-mesenchymal transition (EMT) progression. Finally, Rap1 agonists were utilized to determine the therapeutic mechanism of CLM on cortisol or corticosterone (Cort)-induced EMT progression in lung cancer cells and a mouse lung cancer model.

**Results:**

In our study, CUMS promoted lung cancer xenograft growth, increased the expression of the proliferation marker Ki67, and elevated serum Cort levels. CLM treatment not only alleviated CUMS-induced depression-like behaviors, but also suppressed stress-driven tumor growth. These effects were replicated in a urethane-induced lung cancer model combined with CUMS. Proteomic analysis revealed that CLM’s anti-tumor effects were associated with modulation of the Rap1 pathway. Mechanistically, CUMS downregulated Rap1GAP, activating Rap1 and subsequent ERK1/2 phosphorylation, thereby promoting EMT in lung cancer tissues. CLM effectively reversed these effects by inhibiting Rap1/ERK-mediated EMT. *In vitro*, CLM suppressed cortisol-induced migration, invasion, and EMT in lung cancer cells, and these effects were attenuated by Rap1 agonists. Furthermore, CLM inhibited Cort-induced EMT and depression-like behaviors *in vivo*, while Rap1 activation diminished CLM’s efficacy against Cort-driven tumor growth.

**Conclusion:**

These findings suggest that Rap1/ERK-mediated EMT is a hallmark of chronic stress-associated lung cancer progression. CLM exerts its therapeutic effects by targeting this pathway, offering a novel strategy to mitigate stress-aggravated oncogenesis.

## 1 Introduction

The clinical association between chronic stress and tumor development has become an important focus in cancer research (Cui et al., 2021a). Extensive epidemiological and clinical studies have shown that long-term psychological stress is closely related to an increased risk of various cancers, accelerated progression, and poor prognosis, especially: breast cancer, lung cancer, and colorectal cancer (Walker et al., 2014; Walker et al., 2021). Chronic stress activates the neuroendocrine system and the sympathetic nervous system, leading to the release of stress hormones, such as cortisol, corticosterone (Cort), adrenaline et. cl. These hormones subsequently influence the tumor microenvironment, immune function, and cellular behavior, promoting tumor initiation and metastasis (Ma and Kroemer, 2024). Clinical studies have found that serum cortisol levels are significantly elevated in patients with chronic stress and are associated with tumor aggressiveness and poor prognosis (Yang et al., 2019; Kanter et al., 2024). High levels of cortisol activate the glucocorticoid receptor (GR), promoting tumor cell proliferation, survival, and metastasis, while simultaneously suppressing immune cell function and weakening anti-tumor immune responses (Liu et al., 2022). Additionally, chronic stress can induce the release of catecholamines through the sympathetic nervous system, activating the β-adrenergic receptor signaling pathway and promoting angiogenesis and tumor cell proliferation (Thaker et al., 2006; Zhang et al., 2019).

The relationship between chronic stress and tumorigenesis has become an important area of cancer research, with the activation of the cortisol and its receptor, the GR signaling pathway, considered one of the core mechanisms (Dai et al., 2020). Chronic stress activates the hypothalamic-pituitary-adrenal axis, leading to sustained release of cortisol or Cort, which then regulates tumor cell behavior, the tumor microenvironment, and immune responses through the GR signaling pathway, thereby promoting tumor initiation, progression, and metastasis (Bortolato et al., 2017). Cortisol or Cort binds to GR to directly regulate tumor cell proliferation, apoptosis, and invasion. GR, as a nuclear receptor transcription factor, can enter the cell nucleus and bind to glucocorticoid response elements, regulating the expression of downstream genes (Ma and Kroemer, 2024). Recent studies have reported that chronic stress promotes cortisol release, activates GR signaling, leading to abnormal cholesterol metabolism and ultimately promoting esophageal carcinogenesis (Wang et al., 2025). In addition, Activation of GR signaling promotes the expression of PD-L1 in pancreatic cancer cells, suppresses MHC-I expression, and facilitates immune evasion and resistance to immunotherapy, depletion or pharmacological inhibition of GR in pancreatic cancer cells inhibits the growth of pancreatic tumors (Deng et al., 2021). Meanwhile, Cort can inhibit immune cell function via GR signaling, such as suppressing the activity of natural killer cells and cytotoxic T cells, reducing anti-tumor immune responses, and promoting tumor immune evasion (Wu et al., 2023; Zhang et al., 2024). Furthermore, By increasing plasma Cort, chronic stress impaired type I interferon production in dendritic cells and reduced IFN-γ positive T cell activation (Yang et al., 2019). Although studies have revealed various mechanisms by which chronic stress promotes tumors through the Cort-GR pathway, the specific molecular network remains to be further explored. Targeting the GR signaling pathway may provide new strategies for cancer treatment, especially for patients with stress-related tumors. Future research should focus on the potential of combined psychological interventions and drug treatments in cancer prevention and therapy, offering new approaches to improve patient prognosis.

In oncology, Traditional Chinese Medicine (TCM) offers valuable treatment strategies. It was reported that TCM application could prevent tumorigenesis, attenuate toxicity and enhance the treatment effect, and reduce tumor recurrence and metastasis (Ling et al., 2014). Various monomers and formulas of TCM have been proven to reverse chronic stress-induced tumor progression. The antidepressant Sini San blocks chronic stress-promoted breast cancer stemness via cortisol-dependent GRP78 inhibition, its main component Naringen, inhibits chronic stress-induced breast cancer growth and metastasis by modulating estrogen metabolism through FXR/EST pathway (Zheng et al., 2021; Zhang et al., 2023). Xiaoyaosan slows cancer progression and ameliorates gut dysbiosis in mice with chronic restraint stress and colorectal cancer xenografts (Zhang et al., 2020b). Besides, baicalin suppresses chronic stress-promoted metastasis of breast cancer through direct modulation of the β2-adrenergic receptor, leading to inhibition of the cAMP-PKA signaling cascade and consequent suppression of epithelial-mesenchymal transition (EMT) in breast cancer cells (Jia et al., 2024). Chaihu Longgu Muli Decoction (CLM) is a classical formula prescribed to patients to relieve depression (Zhao et al., 2023). However, the pharmacological basis for CLM’s inhibition of chronic stress-induced lung cancer progression remains uncharacterized.

In this study, we used a mouse ectopic transplant tumor model and a urethane-induced mouse lung cancer model to demonstrate that depression induced by CUMS or Cort promotes lung cancer growth and EMT progression. We further explored the therapeutic effects and mechanisms of CLM on chronic stress-induced lung cancer and investigated related signaling pathways to determine the potential mechanisms of CLM in treating stress-induced lung cancer. Our study not only reveals the potential link between chronic stress and the lung cancer EMT process but also highlights CLM as a potential therapy for lung cancer patients with chronic psychological stress.

## 2 Materials and methods

### 2.1 Drugs and antibodies

Cort (Purity ≥ 98%, C104537), cortisol (Purity ≥ 98%, H657409) and urethane (Purity ≥ 98%, U299635), oxaliplatin (OXA) (Purity ≥ 99%, O124003) were purchased from Aladdin Biotechnology Co., Ltd. (Shanghai, China). 8-CPT-Cyclic AMP (8-CPT) (Purity ≥ 98%, GC15352) was purchased from GLPBio (United States). The β-actin (GB15003), GAPDH (GB15004), Vimentin (GB11192), Rap1GAP (GB111473) and Ki67(GB111141) were purchased from Servicebio (Wuhan, China). E-cadherin (E-cad) (A20798) and N-cadherin (N-cad) (A3045) were purchased from Abclonal (Wuhan, China). The antibody of ERK1/2 (T40071), Phospho-ERK1 (T202/Y204)+ERK2 (T185/Y187) (p-ERK1/2) (T40072), and Ras-related protein-1 (Rap1) (T58240) were obtained from Abmart Biotechnology Co., Ltd. (Shanghai, China).

### 2.2 Preparation of the CLM

In this research, the CLM was composed of the following 10 dried raw herbs: *Bupleurum chinense* DC. (Chai Hu, Hebei, Lot number: 240,701, 12 g). *Os Draconis* (Long Gu, Shanxi, Lot number: 24,012,101, 4.5 g). *Concha ostreae* (Mu Li, Shandong, Lot number: 240,701, 4.5 g). *Scutellaria baicalensis* Georgi (Huang Qin, Shanxi, Lot number: 240,201, 4.5 g). *Panax ginseng* C. A. Mey. (Ren Shen, Jilin, Lot number: 230,902, 4.5 g). *Cinnamomum cassia* (L.) J. Presl (Gui Zhi, Guangxi, Lot number: 23,122,109, 4.5 g). *Poria cocos* (Schw.) Wolf (Fu Ling, Jiangxi, Lot number: 240,601, 4.5 g). *Pinellia ternata* (Thunb.) Makino (Ban Xia, Gansu, Lot number: 20,270,401, 10 g). *Rheum palmatum* L. (Da Huang, Gansu, Lot number: 230,901, 6 g). *Zingiber officinale* Roscoe (Sheng Jiang, Henan, Lot number: 20,240,909, 4.5 g). These herbs were obtained from Beijing Tongrentrang Co., Ltd. (Zhengzhou, China), and certified by experts from Henan University of Chinese Medicine. The voucher specimens were deposited in Herbarium Center of Henan University of Chinese Medicine.

**FIGURE 1:**
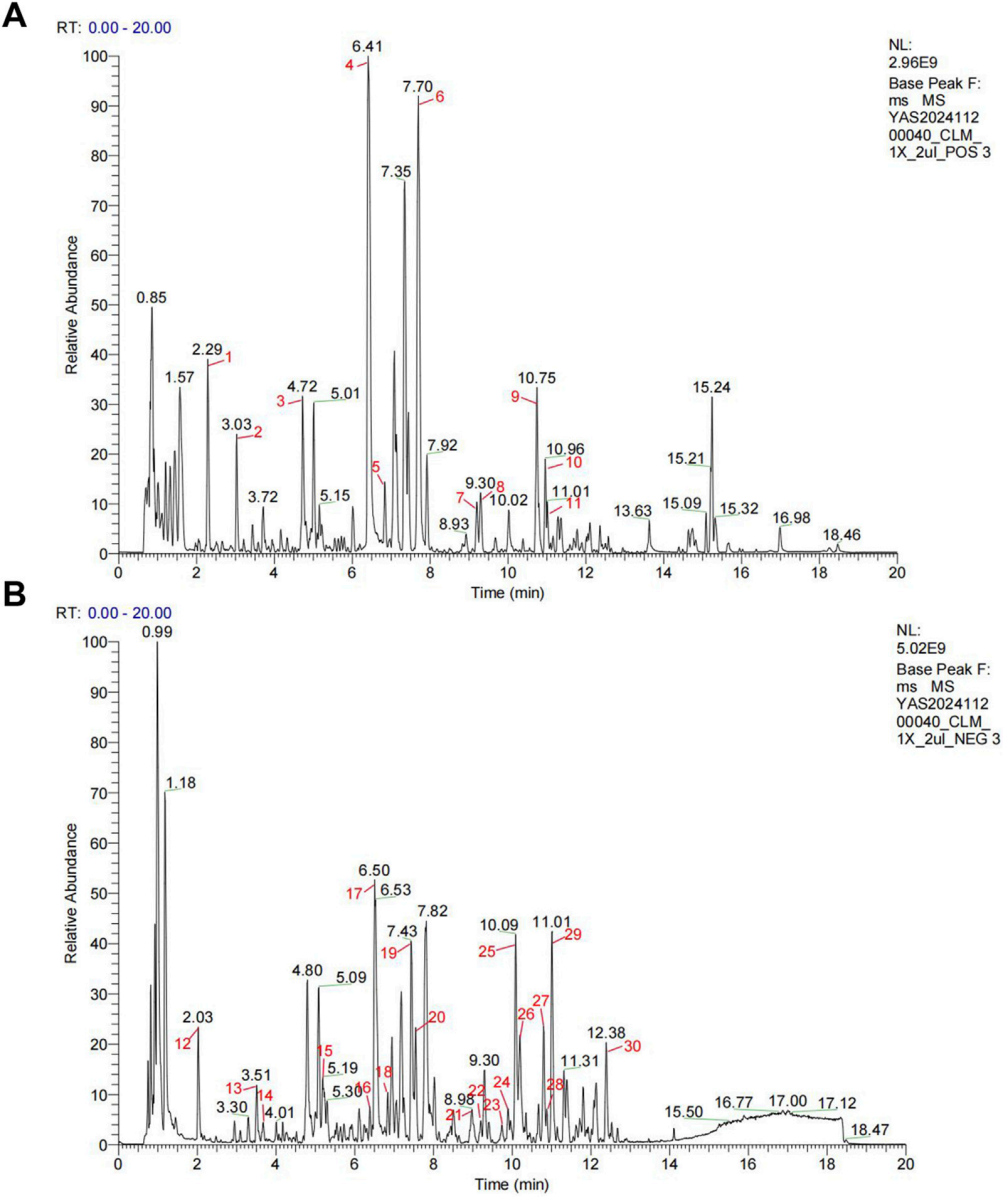
Representative base peak chromatograms of CLM. **(A)** Profile acquired in positive ionization mode. **(B)** Profile acquired in negative ionization mode.

The herbs underwent two rounds of boiling-water extraction. After mixing the two liquid medicines, suspension steaming was carried out to obtain about 1.3 L of drug concentrated solution. Then put it in the refrigerator at −80°C for the night, and put the medicine in the freeze-dryer at −35°C for dehydration the next day to get the freeze-dried powder for experiment. The clinical equivalent dose of CLM was calculated as: CLM = 9.1×(raw herb mass/60 kg) × extraction yield. The CLM powder was dissolved in purified water at three concentrations for animal treatment (0.65, 1.3, and 2.6 g/kg/day, representing low, medium, and high doses, respectively) and administered via oral gavage. Chemical components in CLM were analyzed using Q-Exactive HFX, with base peak chromatograms provided in Figure 1, and the chemical components in CLM were shown in Supplementary Table S1.

### 2.3 Cell culture

The murine Lewis lung carcinoma cell line LLC, along with human lung cancer cell lines A549 and H1299, were obtained from Pricella Biotechnology Co., Ltd. (Wuhan, China). All cell lines were cultured in high-glucose DMEM medium (PM150210, Pricella, Wuhan, China) supplemented with 10% (v/v) fetal bovine serum (PM164210, Pricella) and 1% penicillin-streptomycin (PB180120, Pricella). The cells were maintained in a humidified incubator at 5% CO_2_ at 37°C.

### 2.4 Ethics statement

Six-week-old female C57BL/6J and ICR mice were obtained from Beijing Vital River Laboratory Animal Technology. Following quarantine, the mice were maintained in a specific pathogen-free facility at Henan University of Chinese Medicine, Zhengzhou, China. The animals were housed under controlled conditions (25°C, 55% humidity) with a 12-h light/dark cycle and provided with standard rodent diet and water *ad libitum*. All experimental procedures adhered to the National Institutes of Health’s Guidelines for the Care and Use of Laboratory Animals and were approved by the Experimental Animal Welfare Ethics Committee of Henan University of Chinese Medicine (Approval No. IACUC-S202403124).

### 2.5 CUMS procedure

To induce depression, CUMS was implemented using a combination of eight distinct stressors. These included: 1) restraint in a well-ventilated 50 mL polypropylene tube from 10:00 a.m. to 2:00 p.m.; 2) 24 h food deprivation; 3) 24-h water deprivation, 4) exposure to damp bedding for 24 h; 5) tail pinching for 5 min; 6) forced swimming in 4°C ice water for 2 min; 7) 24-h light/dark cycle reversal, and 8) tilting the cage at a 45° angle for 24 h. Each day, two stressors were randomly applied without repetition to ensure variability and unpredictability in the stress protocol.

### 2.6 Mice lung cancer model and drug administration

For the xenograft tumor mouse study. C57BL/6J mice were randomly divided into five different groups: control (n = 6), CUMS (n = 6), CUMS + CLM-L (n = 6), CUMS + CLM-M (n = 6), and CUMS + CLM-H (n = 6). One week after CUMS exposure, the xenograft tumor mouse model of lung cancer cells was established. Briefly, LLC cells were extensively expanded and collected by trypsin digestion. The cells were resuspended in PBS and injected subcutaneously into mice with 1 × 10^6^ cells per mouse. CLM was administered via oral gavage every day. According to the clinical dosage, the concentrations of CLM administered to mice by oral gavage were set as 0.65 g/kg (low), 1.3 g/kg (median), and 2.6 g/kg (high), respectively. Tumor volume was measured every 2 days and calculated with the formula (width^2^ × length/2). When the maximum tumor volume reaches approximately 1,500 mm^3^, behavioral assessments (open field test and sucrose preference test) were performed. Following sacrifice, serum and tumor tissues were collected for analysis.

For urethane induced lung cancer model, the ICR female mice were received intraperitoneal (i.p.) injections of urethane (dissolved in 0.9% NaCl saline solution, 600 mg/kg of body weight) once per week, for a total of 10 weeks. Then, mice were randomly divided into 6 groups, control (n = 6), CLM (n = 6), CUMS (n = 6), CUMS + CLM (n = 6), CUMS + OXA (n = 6), and CUMS + CLM + OXA (n = 6). The CUMS was sustained exposure for 6 weeks; CLM (1.3 g/kg) was administered via oral gavage every day; OXA (5 mg/kg) (dissolved in 0.9% NaCl saline solution) was administered via intraperitoneal twice a week for 6 weeks. After 6 weeks of CUMS exposure, behavioral assessments (open field test and sucrose preference test) were performed. Following sacrifice, serum and lung tissues were collected for analysis.

For the verification of mechanism, the ICR female mice were received intraperitoneal (i.p.) injections of urethane for a total of 10 weeks. Then, mice were randomly divided into 5 groups, control (n = 6), Cort (n = 6), Cort + CLM (n = 6), Cort+8-CPT (n = 6), and Cort+8-CPT + CLM (n = 6). Cort (20 mg/kg) was subcutaneous injection once a day. 8-CPT (10 mg/kg) was intraperitoneal injection once a day. CLM (1.3 g/kg) was administered via oral gavage every day. After 4 weeks of Cort exposure, behavioral assessments (open field test and sucrose preference test) were performed. Following sacrifice, serum and lung tissues were collected for analysis.

### 2.7 Behavioral analyses

The sucrose preference test was conducted by individually housing mice and providing them with two bottles (one containing pure water and the other with a 1% sucrose solution, for 24 h). To eliminate location bias, the positions of the bottles were alternated every 12 h. Following a 12-h water deprivation period after adaptation, each mouse was randomly offered one bottle of pure water and one bottle of 1% sucrose solution for 24 h, with bottle positions switched every 12 h. Sucrose preference was calculated using the formula: Sucrose preference (%) = sucrose consumption/(sucrose consumption + water consumption) × 100%.

In the open field test, mice were placed in the center of a 100 cm × 100 cm × 50 cm arena and allowed to explore freely for 5 min. After a 1-min adaptation period, their movement trajectories were recorded for the subsequent 5 min using the SMART 3.0 behavioral analysis system.

In the tail suspension test, mice were suspended 1 cm from the tail tip, with immobility time recorded by the SMART 3.0 system during the 5-min trial.

### 2.8 Serum cort detection

After the blood of mice was collected, the serum was obtained by centrifugation for the detection of Cort. The serum Cort content of mice was detected by Mouse Cort ELISA Kit (E-OSEL-M0001, Elabscience Biotechnology Co.,Ltd., Wuhan, China) according to the instructions of the kit manufacturer.

### 2.9 Immunohistochemistry

Tissue samples were fixed in 4% paraformaldehyde for 24 h, followed by paraffin embedding and sectioning into 5 µm slices using a microtome. The sections were mounted on glass slides, dried, and subsequently deparaffinized in xylene. Rehydration was achieved through a graded ethanol series (100%, 95%, 70%). Antigen retrieval was performed enzymatically to unmask epitopes. To minimize non-specific binding, sections were blocked with animal serum. Primary antibodies were applied and incubated overnight at 4°C, followed by secondary antibody incubation at room temperature for 1 h. Color development was conducted using a substrate solution, and nuclei were counterstained with hematoxylin.

### 2.10 Proteomic analysis

4D label free quantitative proteomics was used to analyze the differential proteins in the lung tissues of mice in each group. Control, CUMS, and CUMS + CLM group lung tissues were lysed and quantified. These protein samples were quantitatively analyzed by Shanghai Zhongke Xinsheng Biotechnology Co., Ltd. Bioinformatics tools (http://www.sangerbox.com) were used to analyze the proteome data, identify differentially expressed proteins, and perform KEGG pathway enrichment analysis on the differentially expressed proteins.

### 2.11 qPCR

Total RNA was extracted from lung tissues using the Trizol method. The concentration and purity of the extracted RNA were measured using a spectrophotometer, ensuring that the A260/A280 ratio was between 1.8 and 2.0. The extracted RNA was subjected to reverse transcription using a reverse transcription kit (G3327, Servicebio, Wuhan, China) to convert mRNA into cDNA. A qPCR kit (G3321, Servicebio) was used to prepare the qPCR reaction mixture. PCR amplification was performed by placing the reaction mixture in a real-time PCR instrument and setting the appropriate cycling program. The qPCR instrument recorded fluorescence signals after each cycle, with the fluorescence intensity proportional to the amount of cDNA. The relative expression levels were calculated using the 2^−ΔΔCT^ method to compare mRNA expression differences between different samples or treatment groups. Normalization was performed using the control gene (Gapdh). The primers used in the experiment are listed in Supplementary Table S2.

### 2.12 Western blot

Lung cancer tissues and cells were lysed in RIPA buffer (G2002, Servicebio) containing PMSF (IP0280, Solarbio) and phosphatase inhibitors (P1260, Solarbio). Following centrifugation (12,000 g, 20 min, 4°C), protein concentrations were measured with a BCA kit (PC0020, Solarbio). Equal protein amounts (denatured in 20% loading buffer, 95°C, 5 min) were separated by 8%–15% SDS-PAGE and transferred to PVDF membranes (IPFL00010, Sigma-Aldrich). Membranes were blocked with 5% skimmed milk/TBST (2 h), then incubated with primary antibodies (4°C, overnight). After five TBST washes, HRP-conjugated secondary antibodies (RGAR001, Proteintech) were applied (1 h, RT). Protein bands were detected using ECL reagent (MA0186-1, Meilunbio) and quantified.

### 2.13 Rap1 activity assay

Rap1GTP levels were quantified using the Anti-Rap1GTP Monoclonal Antibody (NewEast Biosciences, 26,912). Mouse lung cancer tissue samples were lysed using IP lysis buffer (Beyotime, P0013) to extract total protein. The lysates were then incubated with an anti-active Rap1GTP antibody coupled to protein A/G agarose beads at 4°C for 3 h under constant rotation to facilitate immunocomplex formation. After incubation, the agarose beads were precipitated by centrifugation, washed three times, resuspended in loading buffer, and denatured by heating at 95°C for 5 min. The samples were subsequently resolved by SDS-PAGE and transferred to PVDF membrane for Western blot analysis to detect the activated Rap1 levels.

### 2.14 Cell viability assay

The cell viability of H1299 and A549 was assessed by MTT assay. Cells were seeded in 96-well plates (6 × 10^3^cells/well, 0.1 mL/well) overnight, then treated with indicated drugs: cortisol (2 μM) or CLM (50 and 100 μg/mL). After 48 h incubation, cells were washed twice with PBS and incubated with 0.5 mg/mL MTT (IM0280, Solarbio) for 4 h at 37°C. The formazan crystals were dissolved in 100 μL DMSO after supernatant removal, and absorbance was measured at 490 nm using a microplate reader.

### 2.15 Wound healing and transwell assay

For wound healing assay, H1299 or A549 cells were seeded in 24-well plates for 12 h before treatment with cortisol (2 μM) or CLM (50/100 μg/mL). After creating a scratch at 0 h, wound closure was quantified at 24 h.

For transwell assay, H1299 or A549 cells (3 × 10^4^/well) in serum-free medium were seeded into 8 μm pore chambers pre-coated with Matrigel (G4131, Servicebio, Wuhan, China), with complete medium in the lower chamber containing the same drug concentrations. After 24 h, invaded cells were fixed, stained with crystal violet, and quantified using ImageJ.

### 2.16 Statistical analysis

All statistical analyses were performed using SPSS 19.0 software. Data are presented as mean ± SEM and visualized using GraphPad 8.0 software. Differences between groups were assessed using a two-tailed, unpaired Student’s t-test, with statistical significance defined as *P* < 0.05.

## 3 Results

### 3.1 CLM improves depression like behavior and inhibits lung cancer transplantation tumor growth induced by CUMS in mice

**FIGURE 2:**
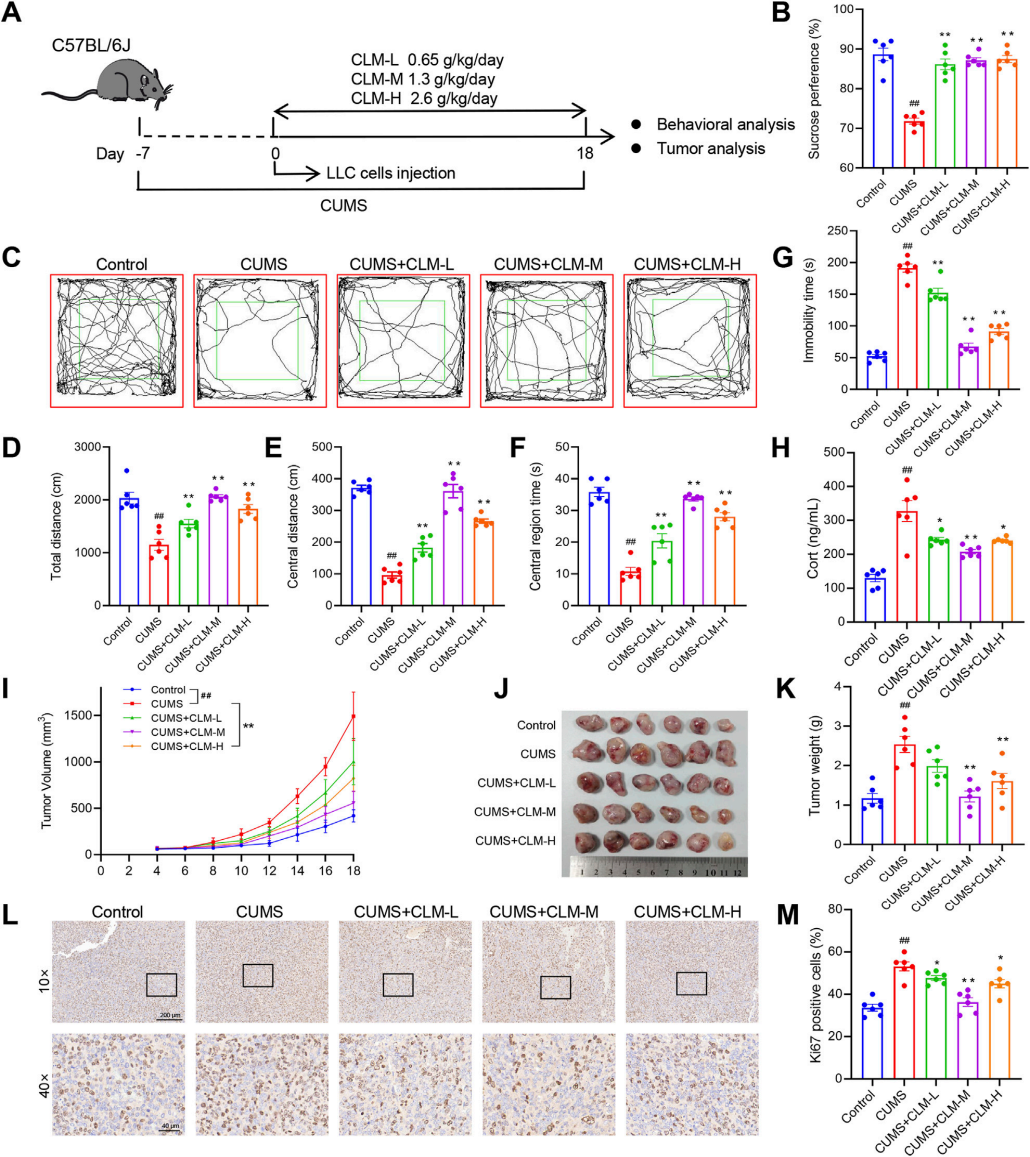
CLM improves depression like behavior and inhibits transplantation tumor growth induced by CUMS in mice. **(A)** Schematic of CUMS exposure of the lung tumor model. **(B)** Sucrose preference of tumor bearing mice (n = 6). **(C)** Trajectory diagram of open field test. **(D)** The total distance of mice in open field (n = 6). **(E)** The central distance of mice in open field (n = 6). **(F)** The central region time of mice in open field (n = 6). **(G)** The immobility time of tail suspension test (n = 6). **(H)** The serum Cort content of mice was detected by the ELISA kit (n = 6). **(I)** Tumor growth curve of tumor bearing mice in different groups. **(J)** Photos of transplanted tumors (n = 6). **(K)** Tumor weight (n = 6). **(L)** Representative photos of Ki67 immunohistochemical staining. **(M)** Statistical analysis of Ki67 positive cell rate (n = 6). Data are represented as mean ± SEM. *P < 0.05, **P < 0.01, compared with CUMS group; ^##^ *P* < 0.01, compared with control group.

We developed the lung cancer mouse model with depression using the protocol outlined in Figure 2A. Behavioral assessments were conducted on the 18th day. Compared with control mice, CUMS-exposed mice exhibited prolonged immobility time, reduced sucrose preference and total distance, indicating that the CUMS model was successfully established (Figures 2B–G). Following treatment with CLM at varying doses, the depression like behavior of mice was significantly improved, as evidenced by the total open field distance, central distance, central residence time and sucrose preference were significantly improved, and the immobility time was shortened, Notably, the most significant improvement in depressive-like behaviors was observed with CLM at a dose of 1.3 g/kg (Figures 2B–G). The tumor bearing mice with CUMS showed an increase of the concentration of serum Cort, while CLM could reduce the concentration of serum Cort (Figure 2H). Analysis of tumor progression revealed that CUMS significantly accelerated the growth of lung cancer xenografts, leading to increased tumor volume and weight, as well as upregulated expression of Ki67, a proliferation marker, in tumor tissues (Figures 2I–M). CLM administration markedly suppressed CUMS-induced tumor growth and Ki67 expression, with the intermediate dose demonstrating the most pronounced efficacy (Figures 2I–M). Collectively, these findings indicate that CLM effectively ameliorates depression like behavior and inhibits transplantation tumor growth induced by CUMS.

### 3.2 CLM improves depression like behavior in urethane induced lung cancer combined with CUMS induced depression mice

**FIGURE 3:**
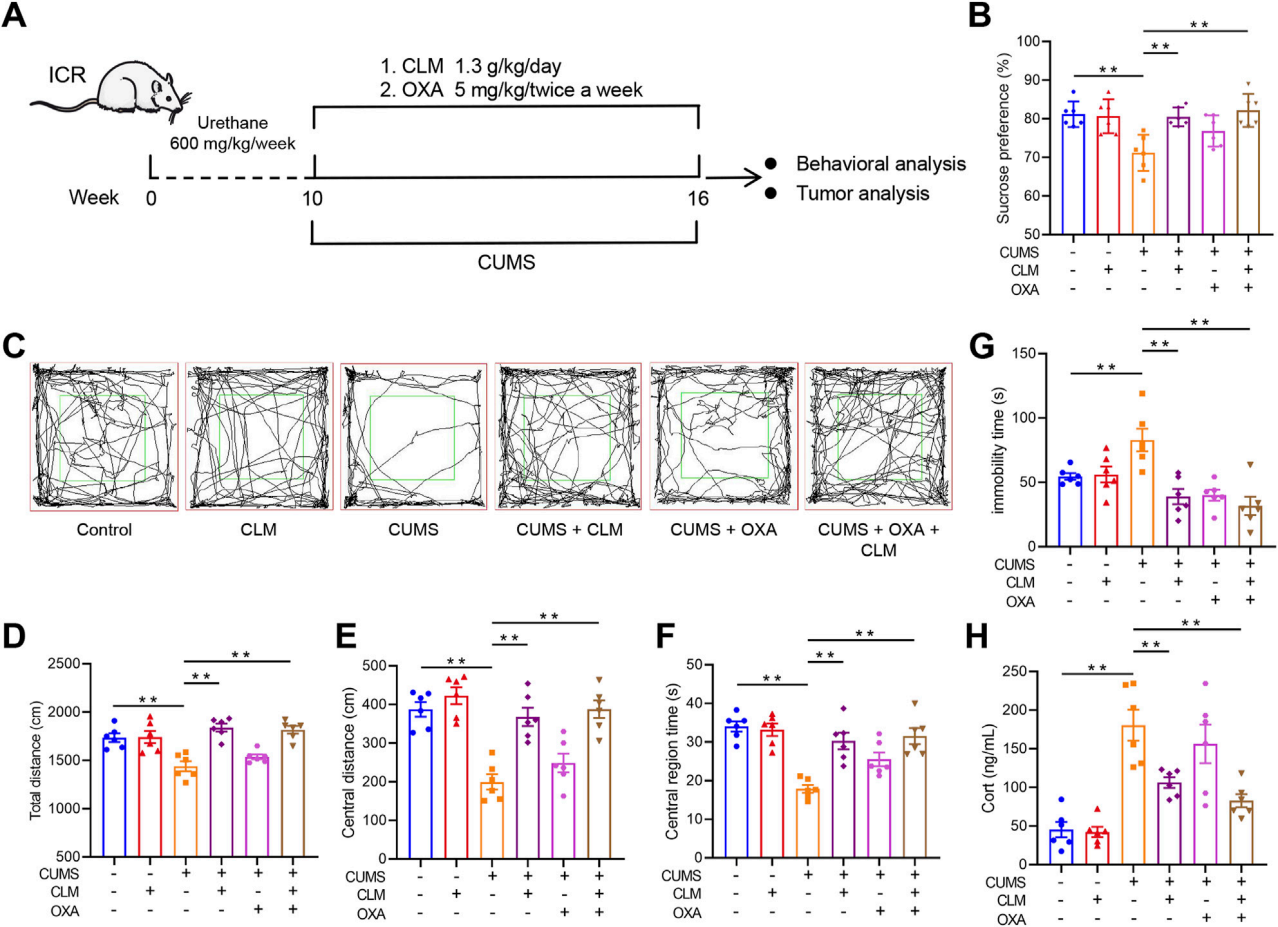
CLM improves depression like behavior in urethane induced lung cancer combined with CUMS in mice. **(A)** Schematic of CUMS exposure of the lung cancer model. **(B)** Sucrose preference of tumor bearing mice (n = 6). **(C)** Trajectory diagram of open field test (n = 6). **(D)** The total distance of mice in open field (n = 6). **(E)** The central distance of mice in open field (n = 6). **(F)** The central region time of mice in open field (n = 6). **(G)** The immobility time of tail suspension test (n = 6). **(H)** The serum Cort content of mice was detected by the ELISA kit (n = 6). Data are represented as mean ± SEM. **P < 0.01.

To further evaluate the inhibitory effect of CLM on lung cancer growth induced by CUMS, we employed a urethane-induced lung cancer model in mice combined with CUMS exposure to validate the efficacy of CLM, as illustrated in Figure 3A. CUMS-treated mice showed increased immobility time, reduced sucrose preference, and decreased total distance compared to controls, confirming successful model establishment (Figures 3B–G). Following CLM treatment, the depression like behaviors of mice was significantly improved, which showed that the total open field distance, central distance, central residence time and sucrose preference were significantly improved, and the immobility time was shortened (Figures 3B–G). The mice in the OXA group, a positive control for lung cancer, did not show significant improvement in depression-like behaviors (Figures 3B–G). Additionally, CUMS-exposed mice displayed elevated serum Cort levels, which were effectively reduced by CLM treatment (Figure 3H). Overall, CLM demonstrated a significant improvement in depression-like behaviors and exhibited potent antidepressant effects.

### 3.3 CLM inhibits lung cancer growth and EMT process induced by CUMS in mice

**FIGURE 4:**
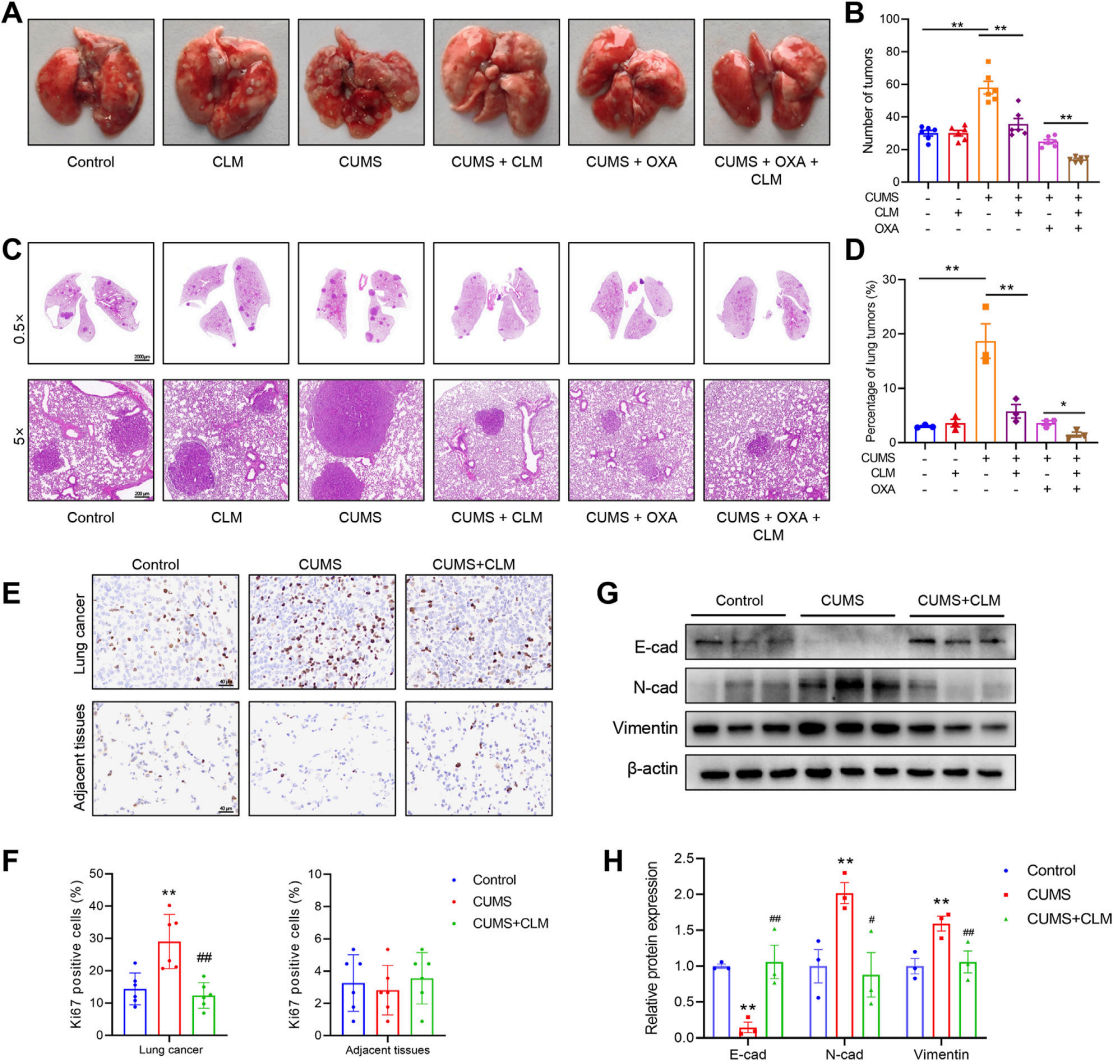
CLM inhibits lung cancer growth induced by CUMS in mice. **(A)** Representative photos of mouse lung tissues. **(B)** Statistical analysis of lung tumor number in mice (n = 6). **(C)** Representative pictures of HE staining of mouse lung tissues. **(D)** Statistical analysis of tumor area in mouse lung tissues (n = 3). **(E)** Representative photos of Ki67 immunohistochemical staining (40×). **(F)** Statistical analysis of Ki67 positive cell rate (n = 6). **(G)** Western blotting analysis of the expression of E-cad, N-cad, and Vimentin in lung tissues (n = 3). **(H)** Statistical analysis of Western blot (n = 3). Data are represented as mean ± SEM. *P < 0.05, **P < 0.01, compared with control group; ^##^ *P* < 0.01, compared with CUMS group.

Compared to the control group, the number of lung tumor nodules and tumor area significantly increased after CUMS exposure (Figures 4A–D). While CLM did not exhibit a therapeutic effect on lung cancer itself, it significantly inhibited tumor growth induced by CUMS (Figures 4A–D). Treatment with the chemotherapeutic drug OXA also led to a significant reduction in the number of lung tumor nodules and tumor area. Furthermore, when combined with CLM, a synergistic anti-tumor effect was observed (Figures 4A–D). Ki67 staining of lung tissue revealed that CUMS exposure promoted Ki67 expression, whereas CLM treatment significantly decreased the number of Ki67-positive cells, with no alteration in Ki67 expression in adjacent normal tissues (Figures 4E,F). EMT is closely related to the occurrence, progression, and metastasis of lung cancer (Menju and Date, 2021). Analysis of key EMT markers demonstrated that CUMS exposure suppressed E-cad expression while promoting the expression of N-cad and Vimentin. In contrast to the CUMS group, CLM significantly inhibited the CUMS-induced EMT process (Figures 4G,H).

### 3.4 CLM attenuates the activation of Rap1/ERK pathway induced by CUMS

**FIGURE 5:**
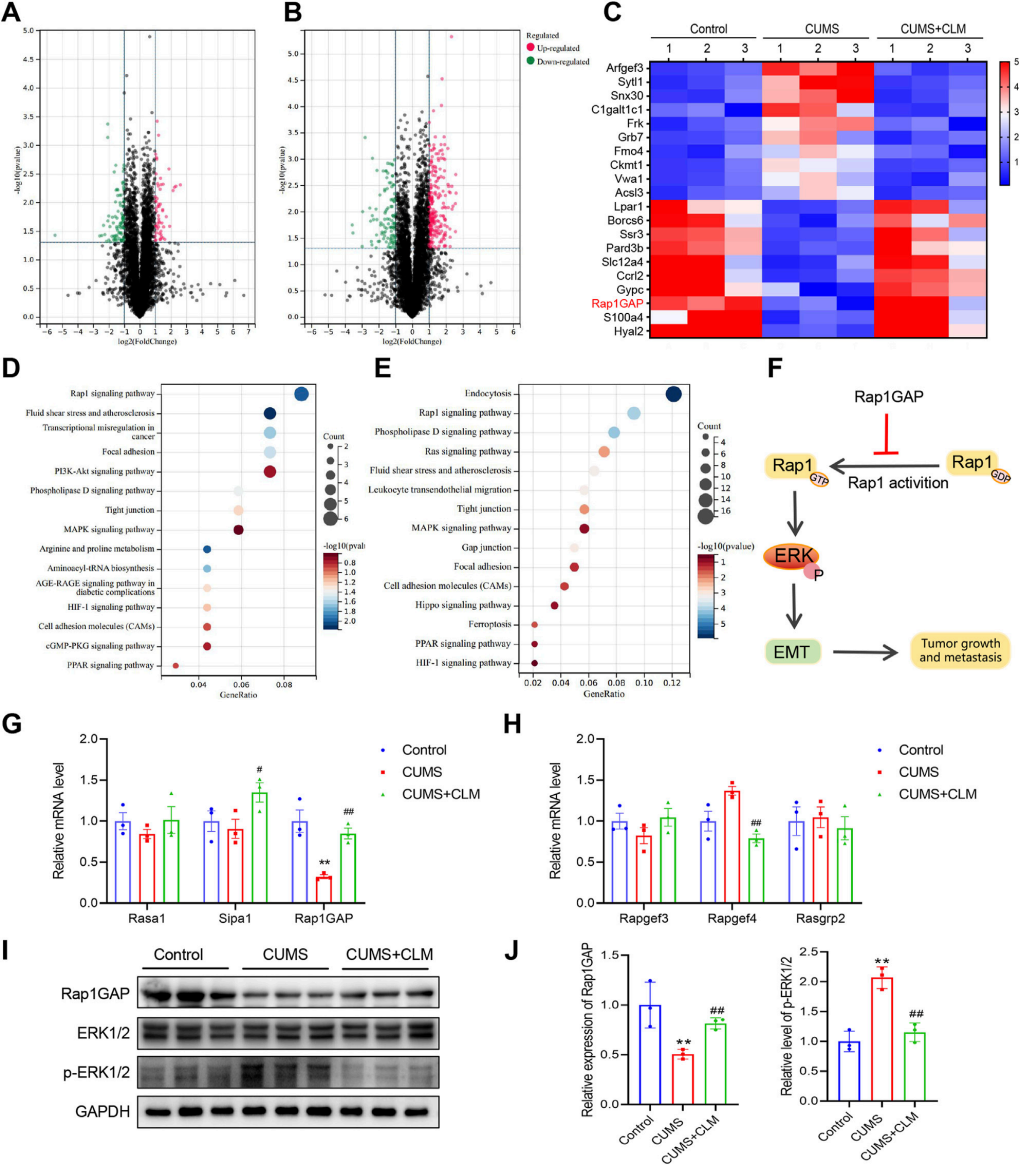
CLM inhibits the activation of Rap1/ERK pathway induced by CUMS. **(A)** The volcanic plots represent the relative expressions of differentially expressed proteins, CUMS group *v. s*. Control group. **(B)** The volcanic plots represent the relative expressions of differentially expressed proteins, CUMS + CLM group *v. s*. CUMS group. **(C)** The heat map of top 20 differentially expressed proteins. **(D)** The KEGG pathway enrichment results of differentially expressed proteins in CUMS and control group. **(E)** The KEGG pathway enrichment results of differentially expressed proteins in CUMS + CLM and CUMS group. **(F)** Schematic diagram of signal pathway transduction. **(G)** The expression of Rap1 repressed related genes was detected by qPCR (n = 3). **(H)** The expression of Rap1 activation related genes was detected by qPCR (n = 3). **(I)** Western blotting analysis of the expression of Rap1GAP, ERK, and p-ERK in lung tissues. **(J)** Statistical analysis of Western blot (n = 3). Data are represented as mean ± SEM. *P < 0.05, **P < 0.01, compared with control group; ^##^ *P* < 0.01, compared with CUMS group.

To further investigate the mechanism by which CLM inhibits stress-induced EMT progression in lung cancer, this study utilized proteomics to identify relevant proteins and signaling pathways involved. Proteomic results showed that 54 proteins were upregulated, and 88 proteins were down-regulated with a ratio value > 2 and *P*-value < 0.05 in the CUMS group compared with the control group (Figure 5A). KEGG pathway analysis of these proteins indicated that the differentially expressed proteins were mainly enriched in the Rap1 signaling pathway, transcriptional misregulation in cancer, phospholipase D signaling pathway, and PI3K-Akt signaling pathway (Figure 5D). Compared with the CUMS group, 233 proteins were upregulated and 102 proteins were downregulated with a ratio value > 2 and *P*-value < 0.05 in the CUMS + CLM group (Figure 5B). KEGG pathway analysis of these proteins demonstrated that the differentially expressed proteins were mainly enriched in the Rap1 signaling pathway, phospholipase D signaling pathway, and Ras signaling pathway (Figure 5E). The top 20 differentially expressed proteins are presented in Figure 5C, including Arfgef3, Sytl1, Rap1GAP, among others. The activation of the Rap1 signaling pathway is closely associated with CLM’s inhibition of stress-induced EMT progression in lung cancer. Rap1 functions as a molecular switch by cycling between the inactive GDP-bound form and the active GTP-bound form. In tumors, activated Rap1 could promote the EMT process by inducing ERK phosphorylation, thereby accelerating tumor growth and metastasis. Rap1GAP is a key inhibitor of Rap1 activation (Shah et al., 2019). The relevant signaling pathway diagram is shown in Figure 5F.

**FIGURE 6:**
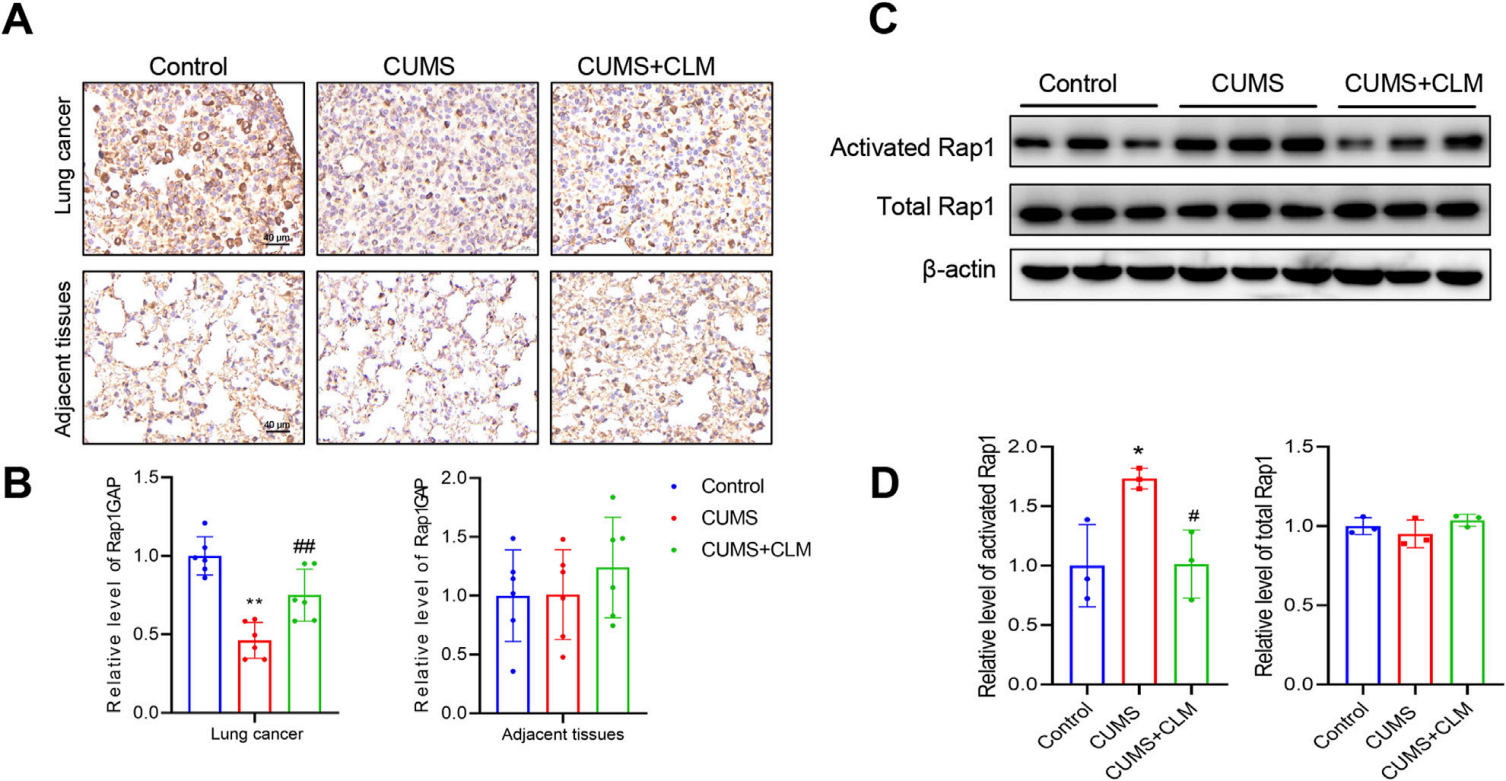
CLM inhibit the activation of Rap1 induced by CUMS. **(A)** Representative photos of Rap1GAP immunohistochemical staining (40×). **(B)** Statistical analysis of Rap1GAP level (n = 6). **(C)** The levels of activated Rap1 and total Rap1 were detected by Western blot (n = 3). **(D)** Statistical analysis of Western blot (n = 3). Data are represented as mean ± SEM. *P < 0.05, **P < 0.01, compared with control group; ^#^ *P* < 0.05, ^##^ *P* < 0.01, compared with CUMS group.

Subsequently, we examined the expression of genes associated with Rap1 inhibition and activation. qPCR results indicated that Rap1GAP was significantly down-regulated in the CUMS group, while CLM was able to restore Rap1GAP expression (Figure 5G). There were no significant changes in Rap1 activation-related genes (Figure 5H). At the protein level, compared to the control group, the level of Rap1GAP was reduced, p-ERK1/2 was increased in the CUMS group, whereas CLM reversed these protein changes (Figures 5I,J). CLM alone showed no significant effect on Rap1GAP expression or ERK1/2 phosphorylation (Supplementary Figure S1). Immunohistochemical staining for Rap1GAP revealed that CLM significantly inhibited the CUMS-induced downregulation of Rap1GAP, while it had no effect on Rap1GAP expression in adjacent non-cancerous tissues (Figures 6A,B). Subsequently, Co-Immunoprecipitation experiments were performed to isolate the active form of Rap1 (Rap1GTP). The results demonstrated that CUMS promoted an increase in Rap1GTP levels in lung cancer, although it did not affect the total amount of Rap1. Importantly, CLM was found to inhibit the activation of Rap1 (Figures 6C,D). In total, CLM attenuates the activation of the Rap1/ERK pathway induced by CUMS.

### 3.5 CLM inhibits cortisol induced migration, invasion, and EMT progression of lung cancer cells, while Rap1 activator reverses the effect of CLM

**FIGURE 7:**
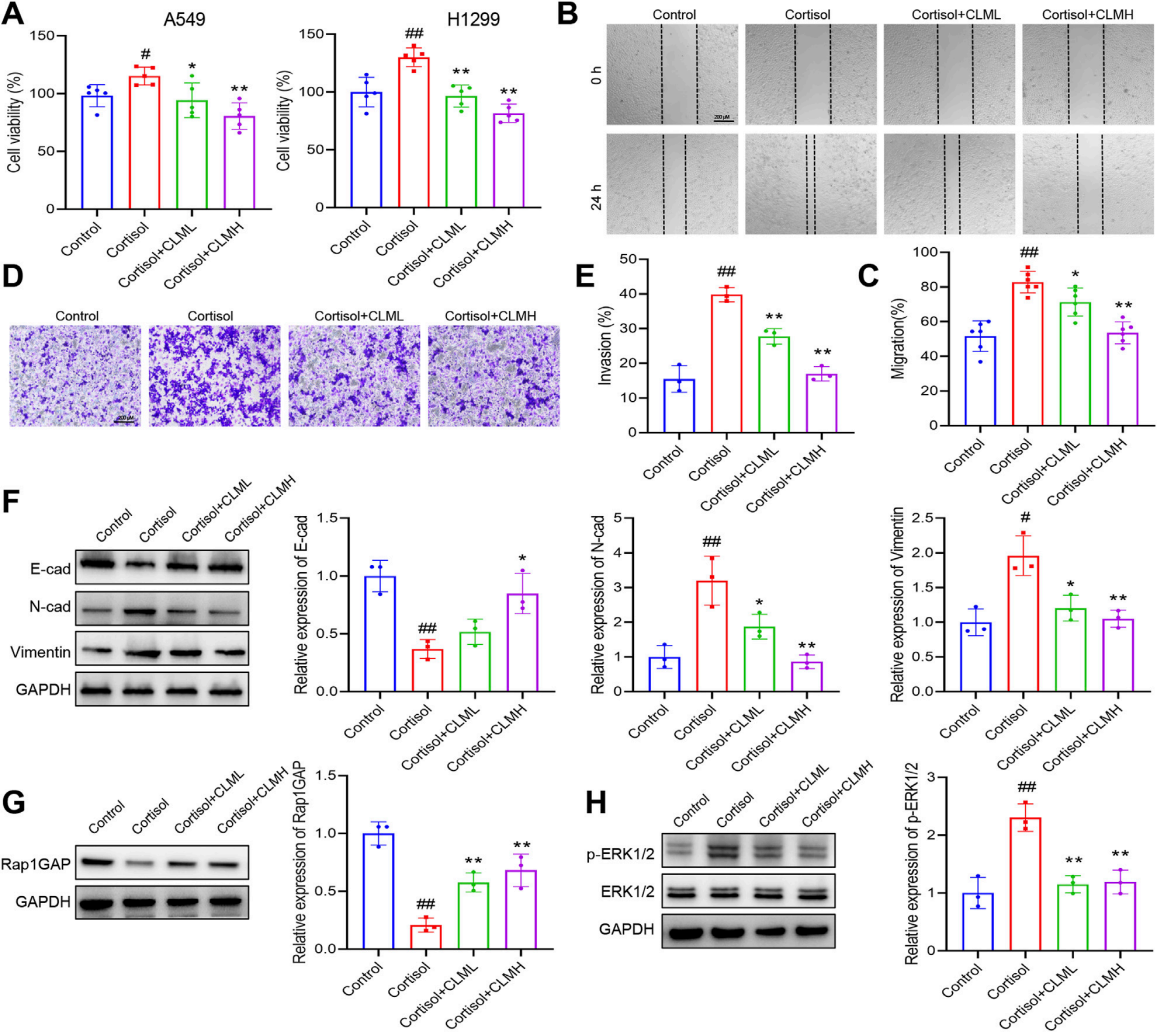
CLM inhibits cortisol induced EMT progression of lung cancer cells. **(A)** The cell viability analysis of A549 or H1299 cells treated with cortisol (2 μM), cortisol (2 μM) + CLML (50 μg/mL), and Cort (2 μM)+CLMH (100 μg/mL) for 48 h (n = 5). **(B,C)** The cell migration ability was assessed using the scratch assay (n = 6). **(D,E)** The cell invasion ability was assessed using the transwell assay (n = 3). **(F)** The protein levels of E-cad, N-cad, and Vimentin in H1299 cells was measured by Western blot (n = 3). **(G,H)** The protein levels of Rap1GAP, ERK1/2, and p-ERK1/2 in H1299 cells was measured by Western blot (n = 3). Data are represented as mean ± SEM. *P < 0.05, **P < 0.01, compared with cortisol group; ^#^ *P* < 0.05, ^##^ *P* < 0.01, compared with control group.

Exposure of cells to cortisol or Cort represents a widely utilized *in vitro* model for studying cellular stress responses (Wang et al., 2025). *In vitro* experiments, lung cancer cells were treated with 2 μM cortisol. The cell viability assay demonstrated that cortisol increased the viability of A549 and H1299 cells, whereas CLM inhibited this cortisol-induced effect (Figure 7A). In the migration assay, CLM effectively suppressed cortisol-induced cell migration (Figures 7B,C), and also reduced cortisol-induced cell invasion in H1299 cells (Figures 7D,E), CLM also showed a consistent effect in A549 cells (Supplementary Figure S2). Analysis of EMT markers revealed that cortisol inhibited E-cad expression, while promoting the expression of N-cad and Vimentin, and CLM was able to reverse these cortisol-induced changes (Figure 7F). Compared to the control group, Rap1GAP expression was reduced, and p-ERK1/2 level was elevated in the cortisol-treated group, whereas CLM reversed these protein alterations (Figures 7G,H).

**FIGURE 8:**
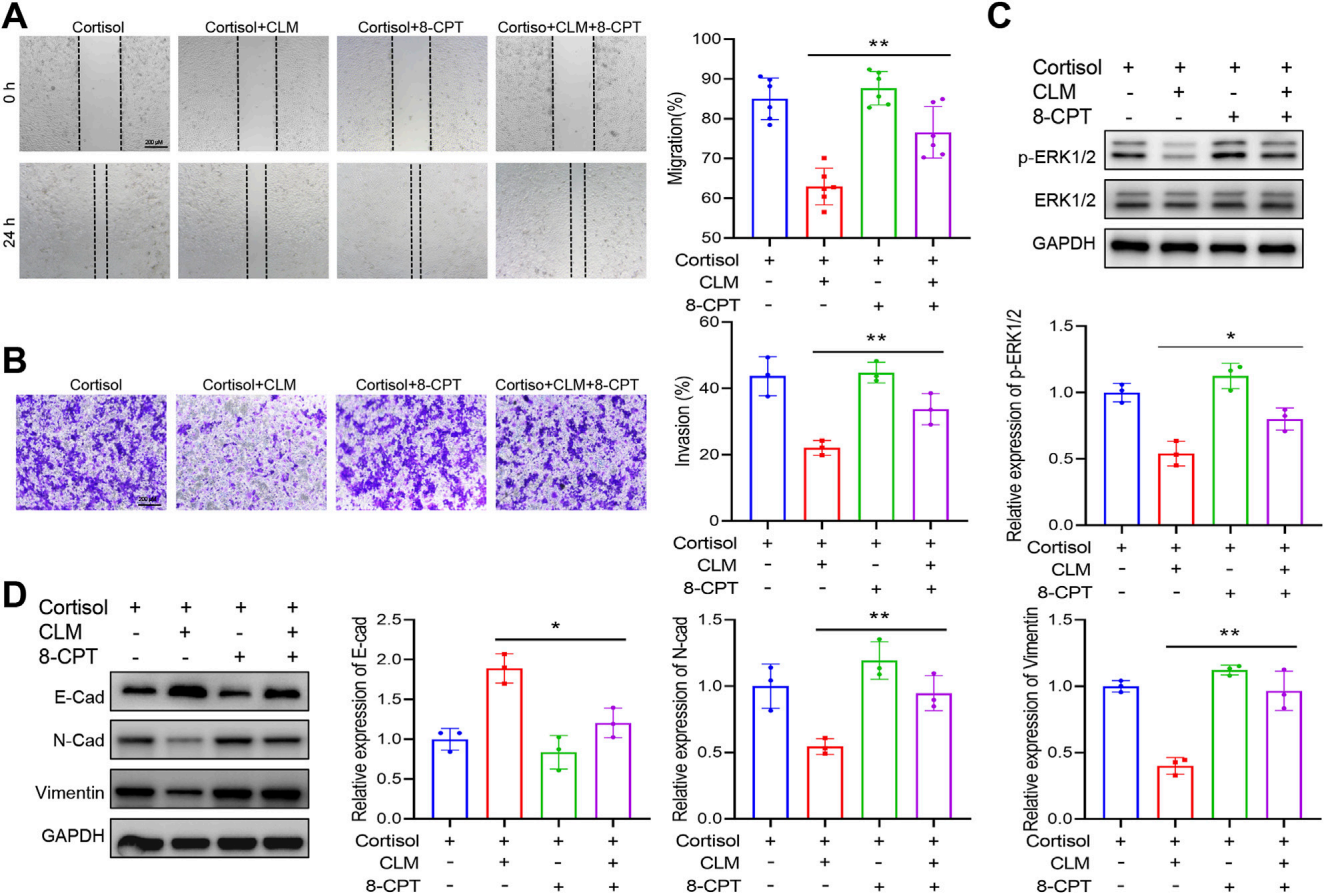
Rap1 activator reverses the effect of CLM on cortisol induced migration, invasion. **(A)** The cell migration ability was assessed using the scratch assay (n = 6). **(B)** The cell invasion ability was assessed using the transwell assay (n = 3). **(C)** The protein levels of ERK1/2, and p-ERK1/2 in H1299 cells was measured by Western blot (n = 3). **(D)** The protein levels of E-cad, N-cad, and Vimentin in H1299 cells was measured by Western blot (n = 3). Data are represented as mean ± SEM. *P < 0.05, **P < 0.01.

To further elucidate the role of Rap1 activation in CLM’s inhibition of cortisol-induced lung cancer progression, a Rap1 activator (8-CPT) was employed. 8-CPT alone did not significantly affect cell migration and invasion but was able to counteract the pharmacological effects of CLM, enhancing cell migration and invasion (Figures 8A,B). Additionally, 8-CPT reversed the downregulation of p-ERK1/2 induced by CLM in the cortisol-treated cell model (Figure 8C). The detection of EMT markers revealed that 8-CPT attenuated the inhibitory effect of CLM on the EMT process (Figure 8D). In total, CLM attenuates the activation of the Rap1/ERK pathway induced by cortisol.

### 3.6 CLM inhibits Cort induced lung tumor growth, while Rap1 activator reverses the efficacy of CLM

**FIGURE 9:**
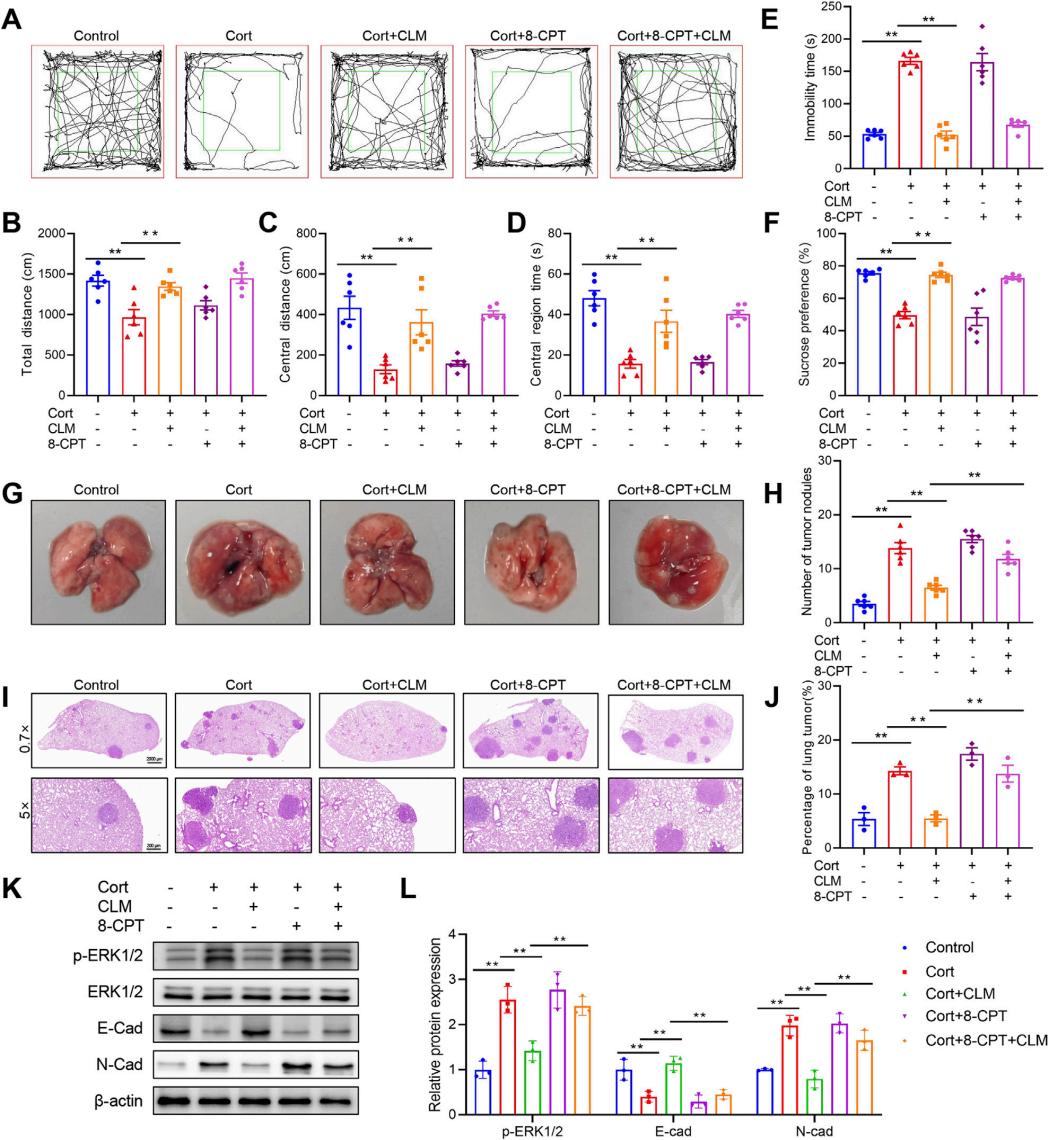
Rap1 activator reverses the efficacy of CLM in the inhibiton of Cort induced lung cancer progress. **(A)** Trajectory diagram of open field test. **(B)** The total distance of mice in open field (n = 6). **(C)** The central distance of mice in open field (n = 6). **(D)** The central region time of mice in open field (n = 6). **(E)** The immobility time of tail suspension test (n = 6). **(F)** Sucrose preference of tumor bearing mice (n = 6). **(G)** Representative photos of mouse lung tissues. **(H)** Statistical analysis of lung tumor number in mice (n = 6). **(I)** Representative pictures of HE staining of mouse lung tissues. **(J)** Statistical analysis of tumor area in mouse lung tissues (n = 3). **(K,L)** Western blotting analysis of the expression of E-cad, N-cad, ERK1/2, and p-ERK1/2 in lung tissues (n = 3). Data are represented as mean ± SEM. **P < 0.01.

Chronic administration of Cort is a well-established method for modeling depression (Zhao et al., 2008). To further explore the mechanism by which CLM inhibits Cort-induced lung cancer progression, we employed a combined lung cancer and Cort stress model. Following 4 weeks of Cort treatment, the open field test results showed that the total distance, center distance, and center time of the Cort group were significantly reduced (Figures 9A–D). The tail suspension test revealed a significant increase in immobility time in the Cort group, and the Cort group also showed a reduced sucrose preference, confirming the successful establishment of the Cort model (Figures 9E,F). Consistent with the CUMS model, the Cort model promoted lung cancer growth, as evidenced by the significant increase in the number and area of lung tumor nodules (Figures 9G–J). CLM not only alleviated Cort-induced depressive-like behaviors but also significantly inhibited Cort-induced lung cancer progression (Figures 9A–J). 8-CPT had no effect on mouse behavior and lung cancer progression, and it did not alter the anti-depressant effect of CLM, but it significantly reversed the anti-tumor effect of CLM (Figures 9A–J). Western blot analysis revealed that CLM reversed the Cort-induced increase in ERK1/2 phosphorylation, and this effect was attenuated by 8-CPT (Figures 9K,L). Furthermore, CLM reversed the Cort-induced EMT process, as evidenced by increased E-cad and decreased N-cad expression. The combined treatment of 8-CPT and CLM diminished the effects of CLM (Figures 9K,L). These findings suggest that the inhibition of Cort-induced lung cancer EMT progression by CLM is closely associated with the modulation of the activation of Rap1/ERK pathway.

## 4 Discussion

Chronic stress plays a significant role in the initiation and progression of tumors. Chronic stress activates the hypothalamic-pituitary-adrenal axis, leading to elevated levels of cortisol. Studies have found that chronic stress-induced increases in cortisol levels suppress immune surveillance, and enhance tumor cell proliferation, invasion, and metastasis by activating the GR signaling pathway. In breast cancer, cortisol inhibits LRP1 expression, and promotes SIRPα expression by GR-dependent transcription, and reduces the phagocytosis of tumor cells by macrophages (Wu et al., 2022). In addition, chronic stress promotes lung metastasis of breast cancer by releasing Cort to activate the formation of neutrophil extracellular traps in lung tissues (He et al., 2024). *In vitro*, it is reported cortisol markedly enhanced the growth of esophageal squamous cell carcinoma cells in a dose-dependent manner (Wang et al., 2025). In lung cancer, stress elevated plasma Cort levels and upregulated the expression of glucocorticoid-inducible factor Tsc22d3, leading to the suppression of T cell activation and impairing therapy-induced antitumor immunity (Yang et al., 2019). Several studies have shown that chronic stress can promote the growth of lung cancer (Yang et al., 2021; Zhou et al., 2024). In a lung cancer xenograft mouse model, CUMS upregulated PD-L1 expression, impaired CD8^+^ T cell cytotoxicity, and elevated MMP2, MMP9, and VEGF levels (Cui et al., 2021b). Our study found that both CUMS and Cort could promote lung cancer growth, increase Ki67 expression, and enhance the EMT process. In addition, our results showed cortisol promotes lung cancer cell migration and invasion *in vitro*. Our study provides more evidence that chronic stress promotes tumor progression.

Rap1, a small GTPase protein, is pivotal in cell signaling, particularly in modulating cell adhesion, migration, and proliferation. Emerging evidence indicates that Rap1 facilitates tumor EMT through the activation of the ERK1/2 signaling pathway, thereby enhancing tumor invasion and metastasis (Looi et al., 2020). A hallmark of EMT is the diminished expression of E-cad, which impairs epithelial junction integrity and contact-mediated growth suppression (Lu and Kang, 2019). Rap1GAP is an important inhibitor of Rap1 activation, could convert it to the inactive GDP-bound state. Previous findings indicate that Rap1GAP acts as a tumor suppressor in multiple cancers (Tamate et al., 2017; Faam et al., 2021). Recent findings have demonstrated a positive correlation between Rap1GAP upregulation and E-cad expression in gastric cancers (Tsygankova et al., 2013; Yang et al., 2017). Mechanistically, Rap1A drives EMT by activating ERK, MAPK, and Notch signaling (Lu et al., 2016). Additionally, studies in breast cancer have revealed that Rap1GAP depletion results in decreased E-cad levels and ERK activation, correlating with increased tumor invasiveness (Shah et al., 2018). It has also been reported that Mex3a overexpression leads to Rap1GAP downregulation, thereby activating the MEK/ERK pathway and promoting oncogenesis in colon cancer (Li et al., 2021). These findings indicate that Rap1GAP downregulation promotes tumor aggressiveness by suppressing E-cad and inducing EMT. In our study, proteomic analysis revealed that chronic stress downregulates Rap1GAP, thereby activating the Rap1 signaling pathway and promoting ERK1/2 phosphorylation and EMT progression. Both *in vitro* and *in vivo* studies have demonstrated that Cort and cortisol can activate the Rap1/ERK signaling pathway. However, the precise molecular mechanisms underlying GR-mediated regulation of Rap1/ERK activation remain incompletely understood and warrant further investigation.

TCM possesses unique advantages in treating depression-related tumor progression, primarily by regulating the body’s overall state and improving psychological and physiological functions, thereby achieving the goal of preventing and treating tumors (Zhang et al., 2020a). Certain TCM formulations exhibit significant antidepressant effects and can reverse tumor growth induced by chronic stress, such as Sini San, Xiaoyao San et. cl. CLM, a classic antidepressant formula, is widely used in clinical settings for the treatment of depression (An et al., 2022; Zhao et al., 2023). It exerts its anti-depressant effects by up-regulated brain-derived neurotrophic factor in the hippocampus (Wang et al., 2019). Our research has found that while CLM does not have a significant therapeutic effect on lung cancer, it could notably inhibit the progression of lung cancer induced by CUMS or Cort. Compared to studies employing single tumor models, we utilized a mouse lung cancer cell LLC transplant tumor model and a urethane-induced lung cancer model, simulating chronic stress with CUMS or Cort. This approach allowed us to systematically demonstrate the pharmacological effects of CLM, showing that it not only alleviates depressive-like behaviors in mice but also exerts a beneficial therapeutic effect on lung cancer mice under chronic stress conditions. Mechanistically, we have discovered that CLM upregulated Rap1GAP, and inhibited the activation of Rap1/ERK signal pathway, thereby suppressing chronic stress-induced EMT progression in lung cancer. Our work reveals the previously unrecognized association between chronic stress and Rap1/ERK pathway activation in lung cancer, establishing a new mechanistic framework for understanding how depression facilitates tumor development. In China, the CLM and its derivatives are widely employed for managing cancer-associated depression, showing considerable effectiveness in enhancing patients’ quality of life Nevertheless, the absence of large-scale clinical studies assessing its therapeutic use and safety parameters hinders broader acceptance in the international medical community. In the future, we will conduct more in-depth research, such as exploring the pharmacological substance basis and action targets of CLM, as well as verifying the effectiveness of CLM in other cancer models.

## 5 Conclusion

Taken together, our findings underscore the pivotal involvement of Rap1/ERK signaling in the EMT process in lung cancer under chronic stress conditions. Notably, CLM exhibits dual therapeutic effects, effectively alleviating depression and suppressing chronic stress-induced EMT in lung cancer by downregulating Rap1GAP and inhibiting the Rap1/ERK pathway activation. This study offers substantial preclinical evidence for the therapeutic potential of CLM in managing lung cancer patients experiencing persistent stress.

## Data Availability

The original contributions presented in the study are included in the article/Supplementary Material, further inquiries can be directed to the corresponding authors.
