# Supplementary material for: Chaihu Longgu Muli Decoction inhibits chronic stress-induced lung cancer epithelial-mesenchymal transition process by suppressing Rap1/ERK signal pathway
Source: Front Pharmacol. 2025 Jul 25;16:1644315. doi: 10.3389/fphar.2025.1644315 (PMC12332513; doi:10.3389/fphar.2025.1644315)
Supplement: Supplementary file 1 [file DataSheet1.docx]

**Supplementary materials**

Supplementary Table 1 Identification of chemical components corresponding to characteristic peaks in base peak chromatogram.

| **No** | **m/z** | **RT/min** | **ppm** | **Adduct** | **Score** | **Compound name** | **SuperClass** |
| --- | --- | --- | --- | --- | --- | --- | --- |
| 1 | 166.0861 | 2.28 | 1.4 | [M+H] | 0.9994 | Phenylalanine | Small peptides |
| 2 | 205.0969 | 3.02 | 1.8 | [M+H] | 0.9695 | Tryptophan | Small peptides |
| 3 | 549.1599 | 4.74 | 0.9 | [M+H] | 0.9537 | 5,7-dihydroxy-2-phenyl-6-[3,4,5-trihydroxy-6-(hydroxymethyl)oxan-2-yl]-8-(3,4,5-trihydroxyoxan-2-yl)-4H-chromen-4-one | Flavonoids |
| 4 | 447.092 | 6.44 | 1.2 | [M+H] | 0.9997 | Baicalin | Flavonoids |
| 5 | 449.1075 | 6.85 | 0.9 | [M+H] | 0.8431 | Naringenin-4'-O-.beta.-D-glucuronide | Flavonoids |
| 6 | 461.1075 | 7.71 | 0.2 | [M+H] | 0.9997 | Oroxindin | Flavonoids |
| 7 | 271.0599 | 9.21 | 0.6 | [M+H] | 0.9996 | Baicalein | Flavonoids |
| 8 | 163.0752 | 9.3 | 1.1 | [M+H] | 0.8878 | 2-Methoxycinnamaldehyde | Phenylpropanoids |
| 9 | 375.1072 | 10.75 | 1.0 | [M+H] | 0.953 | Neobaicalein | Flavonoids |
| 10 | 277.1796 | 10.96 | 1.5 | [M-H_2_O+H] | 0.9776 | (5r)-5-hydroxy-1-(4-hydroxy-3-methoxyphenyl)decan-3-one | NA |
| 11 | 285.0756 | 11.01 | 0.8 | [M+H]+ | 0.9918 | Oroxylin | Flavonoids |
| 12 | 169.0136 | 2.05 | 4.5 | [M-H-C2H4]- | 0.9995 | Ethyl gallate | Phenolic acids (C6- C1) |
| 13 | 289.072 | 3.48 | 0.5 | [M-H]- | 0.9768 | (+)-catechin hydrate | Flavonoids |
| 14 | 175.0605 | 3.65 | 4.3 | [M-H]- | 0.9302 | 2-Isopropylmalic acid | Fatty Acids and  Conjugates |
| 15 | 301.0349 | 5.16 | 1.0 | [M-H]- | 0.9758 | Viscidulin I | Flavonoids |
| 16 | 187.0971 | 6.35 | 2.9 | [M-H]- | 0.9985 | Azelaic acid | Fatty Acids and  Conjugates |
| 17 | 891.162 | 6.48 | 1.3 | [2M-H]- | 0.9886 | Apigenin 7-glucuronide | Flavonoids |
| 18 | 845.4913 | 6.8 | 0.8 | [M+HCOO]- | 0.8023 | Ginsenoside A2 | Triterpenoids |
| 19 | 919.1929 | 7.41 | 1.4 | [2M-H]- | 0.9516 | Oroxyloside | Flavonoids |
| 20 | 475.0882 | 7.5 | 1.4 | [M-H]- | 0.9377 | Hispidulin 7-glucuronide | Flavonoids |
| 21 | 269.0454 | 8.94 | 0.4 | [M-H]- | 0.9973 | Norwogenin | Flavonoids |
| 22 | 431.1707 | 9.17 | 0.9 | [M-H]- | 0.9651 | Ncgc00385574-01![5-acetyloxy-1,7-bis(3,4-dihydroxyphenyl)heptan-3-yl] acetate | Diarylheptanoids |
| 23 | 169.0864 | 9.7 | 4.3 | [M-H-H2O]- | 0.9956 | 3-Isobutylglutaric acid | Fatty Acids and  Conjugates |
| 24 | 971.5227 | 9.87 | 1.0 | [M+HCO2]- | 0.9487 | Saikosaponin C | Triterpenoids |
| 25 | 329.2336 | 10.06 | 5.6 | [M-H]- | 0.9929 | FA 18:1+3o | Octadecanoids |
| 26 | 297.0405 | 10.17 | 1.6 | [M-H]- | 0.9869 | 3,8-Dihydroxy-1-methylanthraquinone-2-carboxylic acid | Polycyclic aromatic polyketides |
| 27 | 373.093 | 10.78 | 1.1 | [M-H]- | 0.9096 | 5-Hydroxy-2-(3-hydroxy-4,5-dimethoxyphenyl)-3,7-dimethoxychromone | Flavonoids |
| 28 | 329.2332 | 10.87 | 1.0 | [M-H]- | 0.997 | (Z)-9,10,11-Trihydroxy-12-octadecenoic acid | Octadecanoids |
| 29 | 283.0249 | 10.99 | 0.9 | [M-H]- | 0.9589 | Rhein | Polycyclic aromatic  Polyketides |
| 30 | 269.0454 | 12.38 | 1.5 | [M-H]- | 0.9998 | Emodol | Polycyclic aromatic  polyketides |

Supplementary Table 2. Sequence (5'-3') of primers used for real time qPCR.

| **Gene** | **Forward (5′ → 3′)** | **Reverse (5′ → 3′)** | **Amplicon size** |
| --- | --- | --- | --- |
| *Rasa1* | TGTGGTGATTACTACATTGGTGG | CGCCTTCTATCTTCTACTGGCTC | 143 |
| *Sipa1* | CCTACTCGGCTCTTCACTGAC | AGCAAGCTCTGCACATCATAG | 122 |
| *Rap1GAP* | ATGGATGAACAGCGATGCTCC | AAACTGGGGCAGTAGGATGAG | 126 |
| *Rapgef3* | TCTTACCAGCTAGTGTTCGAGC | AATGCCGATATAGTCGCAGATG | 223 |
| *Rapgef4* | CAAGGAGAATGTCCCTTCAGAGA | CCGCGAGTGAACACAGGAT | 184 |
| *Rasgrp2* | GCAAAGAGCGCCTGTCAGT | GTGGATGTCAAACACTCCGTC | 203 |
| *Gapdh* | AGGTCGGTGTGAACGGATTTG | TGTAGACCATGTAGTTGAGGTCA | 123 |


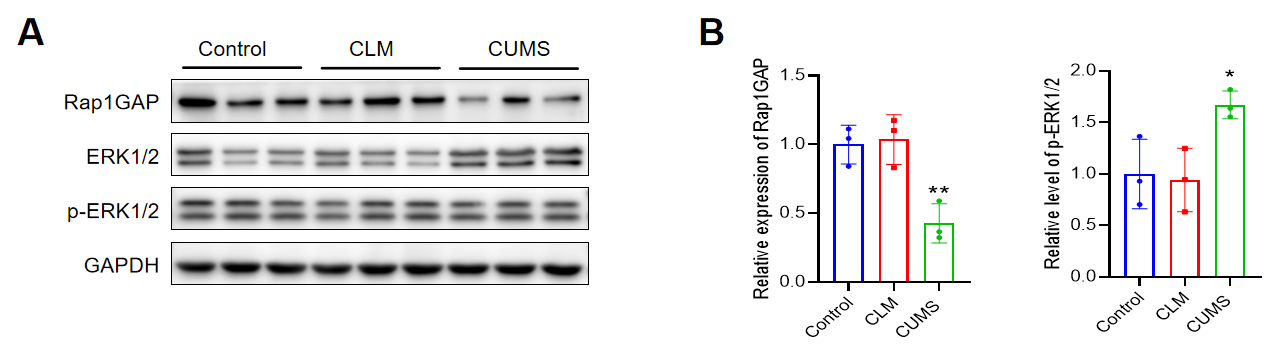


Supplementary Figure 1. (A) Western blotting analysis of the expression of Rap1GAP, ERK1/2, and p-ERK1/2 in lung tissues (n=3). (B) Statistical analysis of western blot (n=3). Data are represented as mean ± SEM. **P* < 0.05, ***P* < 0.01, compared with control group.


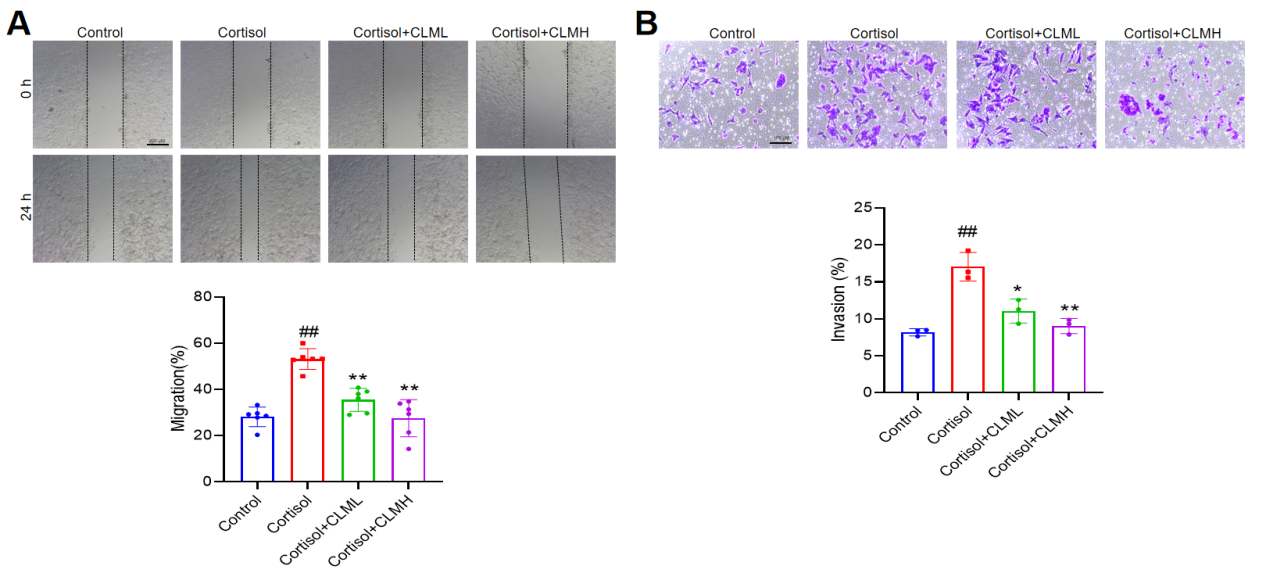


Supplementary Figure 2. (A) The cell migration ability of A549 cell was assessed using the scratch assay (n=6). (B) The cell invasion ability of A549 was assessed using the transwell assay (n=3). Data are represented as mean ± SEM. **P* < 0.05, ***P* < 0.01, compared with cortisol group; ^#^*P* < 0.05, ^##^*P* < 0.01, compared with control group.
